# Continuing medical education programs for primary care physicians from remote locations of Vietnam: a needs assessment

**DOI:** 10.1186/s12909-022-03336-4

**Published:** 2022-04-13

**Authors:** Pham Ngan Giang, Matthew Kelly, Nguyen Thi Tuyet Nhung, Haribondhu Sarma

**Affiliations:** 1 Hanoi Medical University, Hanoi, Vietnam; 2 Research School of Population Health, The Australian National University, 62 Mills Road, Canberra, 2601 Australia

**Keywords:** Continuing Medical Educational, Primary health care, Vietnam, Knowledge, Medical emergency care

## Abstract

**Background:**

Inadequate attention has been given to ensuring ongoing training to improve knowledge, skills and capacity of primary health care providers in low- and middle-income countries. The Hanoi Medical University, Vietnam is providing training sessions for physicians working in commune health stations (CHSs) in three mountainous, remote northern provinces in 2019. This article aims to assess these physicians’ knowledge of correct medical responses to emergencies in order to assess their training needs.

**Methods:**

We conducted a cross-sectional study amongst doctors posted to CHSs located in 3 mountainous remote provinces of northern Vietnam. We used a self-administered questionnaire that comprised questions on common medical emergencies, maternal and child care, and non-communicable disease management. We performed Chi-square tests to assess the statistical significance of differences in the mean proportions of correct answers for each health care question category, and for differences in mean proportions of correct answers by doctor characteristics.

**Results:**

In total 302 doctors were recruited to the study. More than half of the sample answered 30–50% of the questions correctly, followed by around a third who answered 50–70% correctly. Less than 2% of doctors answered more than 70% correct responses to the entire question set. There were statistically significant differences between question categories, with cardiovascular care questions answered correctly significantly less often than any of the categories (*p* < 0.00001).

**Conclusion:**

The findings reported here show that the doctors who participated in the study have relatively low knowledge on common emergencies, particularly to answer cardiovascular care questions. The results also support the need for continuing medical education to improve doctors’ knowledge, who are mostly practicing in resource limited remote settings.

**Supplementary Information:**

The online version contains supplementary material available at 10.1186/s12909-022-03336-4.

## Background

Primary health care (PHC) is the first contact point of the individual, families and societies in health systems [1, 2]. In many low-and-middle income countries, PHC providers provide a range of services including identifying and controlling prevailing health problems, maternal and child health care, family planning, immunization, treatment of common diseases, emergency health services and provision of essential drugs [1]. The providers in PHC facilities also provide regular and wide-ranging clinical management including diagnosis and referral to specialised service providers and hospitals [3]. Despite the wide contribution of PHC providers, inadequate attention has been given to ensuring ongoing training to improve their knowledge, skills and capacity, particularly in remote, hard-to-reach and mountainous areas in low- and middle-income countries, including in Vietnam.

In Vietnam, health care is provided through a combination of public and private health systems. The public system performs an important role in preventive and curative health care services for the population, through specialised tertiary health care hospitals and professionals as well as community level PHC services [4]. Primary health services are operated by Commune Health Stations (CHSs), which have been considered as the foundation of the nation’s health system and playing a very critical role in implementing national health programmes at the community level. The CHSs provide very basic health care services along with initial diagnoses, treatments, and referrals to public hospitals [4]. Each CHS is equipped with a Primary Care Physician (PCP) along with midwife, nurse, assistant doctor of traditional medicine or pharmacist to serve an average 8,000 population in its catchment area. Currently, more than 11,000 CHSs operate in Vietnam, distributed across the 63 provinces located in the six broad geographical regions of the country [5].

In Vietnam, the doctors training program includes six-years of university study. After graduating from the universities, the doctors who are working in large hospitals also should have 3 years of residency training or a 2 years’ master program. Additionally, the doctors working in a large hospital are continuously engaged in several continuing medical education (CME) courses and have opportunities to participate in seminars and different scientific activities. However, the CHSs doctors have been recruited right after their six years of graduation and they do not have opportunities to get further training or participate in any CME except a few targeted national training programs i.e., training on HIV/AIDS. On the other hand, it is very challenging to organize CME programs for commune doctors, because a CHS usually has only five medical staff, of which only one person is a doctor in charge of emergency work and medical examination and treatment for the whole community. Therefore, the professional knowledge of commune doctors is mostly from 6 years of university study, work experience, and self-reading.

Despite a well-structured health care system at the community level, the quality of PHC services provided through the CHSs remains a matter of concern [6]. Health service quality provided through less-qualified health staff at CHSs has been apparent for several decades, mainly because of decline of public funding for human resources development [7]. The quality of the health workforce in CHSs in the diagnosis and management of some common diseases and emergencies is limited due to inadequate professional knowledge, ability to assess patients’ situation and skills in clinical practice [8]. Previous study identified a range of factors both on the supply and demand sides that associated with health service quality at CHSs in Vietnam, including contextual factors such as limitations in availability of medicines and other clinical resources, geographical locations (mountainous and remote areas) which create challenges in access to health care, and limited support skilled workforce are also very critical to consider when assessing the quality of health services [6].

To address these challenges, Vietnam’s government had undertaken a Master Plan for Health Workforce Development from 2012–2020, which had set out comprehensive measures with a focus towards increasing the number of health workers and improving the quality of care through training and education programs [9]. The Master Plan also provided special attention to capacity building in human resources management and staff retention in remote and mountainous areas, resulted increased doctors per 1,000 population [10] and more than 80% of health care providers in CHSs have received at least one in-service training session in the past two years [11]. Despite such findings, there are frequent reports that the existing training and approaches for human resource development for CHSs in Vietnam are not adequate, which highlights the need for targeted training [12–14]. Therefore, the national Health Workforce Development Master Plan has been renewed to cover the next 10-year period (2020–30) [10]. The Master Plan envisages that recurrent training including short courses on day-to-day clinical management, and graduate and post-graduate level education focusing on primary health care may enable health care providers at CHSs to be updated with cutting-edge knowledge that would strengthen their skills and abilities [10].

To achieve the goals of the Health Workforce Development Master Plan the Vietnamese Ministry of Health and Hanoi Medical University has developed a training curriculum for physicians. As part of this training program a pre-test questionnaire was conducted as a knowledge assessment tool. This article reports on the results of this pre-training assessment and aims to measure Vietnamese physicians’ knowledge regarding correct medical responses to general emergencies. The findings are discussed within the context of identifying the most important training needs of CHS physicians in these topical areas of primary care, as well as the options and modalities that can be employed to implement such training as part of Continuing Medical Education (CME) programs across the country which form part of the National Health Workforce Development plan.

## Methods

### Study design, setting and population

The Hanoi Medical University (HMU) was assigned with the responsibility to prepare a training curriculum for physicians as part of the National Health Workforce Development Master Plan and pilot test these training activities in a sample of provinces of Vietnam. As part of this initiative, the HMU provided eight training sessions for CHS physicians from three mountainous, remote northern provinces in 2019. It was envisaged that the experiences and lessons learnt from this initial phase would help develop the national strategy for skill development among CHS physicians. This cross-sectional study was conducted amongst doctors posted to CHSs located in 3 mountainous remote provinces of northern Vietnam. Doctors from these provinces were selected to participate in the HMU Continuing Medical Education (CME) training program. These three provinces contain a population of 2,238,800 people and are serviced by 487 CHS and 426 doctors. Of these doctors, 302 participated in the training program. A total of 8 training programs were conducted during 2019, and included a pre-test questionnaire that was administered to all participants at the commencement of the actual training program.

### Study materials

The overall questionnaire comprised a general component with questions on the basic tasks and responsibilities of CHS physicians, and a clinical component which had three sections on common medical emergencies, maternal and child care, and non-communicable disease management. A draft version of the questionnaire was first piloted in a sample of 20 CHS physicians, working in areas outside those investigated in this study, and then revised to improve the content, style and logical flow of the various items.

In the questionnaire the participants were presented with questions regarding 27 common medical emergencies encountered in primary health care and asked what they considered to be the appropriate medical response to that emergency. The questions were presented in multiple choice format with doctors asked to choose the single most appropriate response. The topic areas covered in the questionnaire were assigned based on the common disease and health patterns in the community and the responsible functions of the Commune Health Stations. The correct answers for each question were assigned based on the instructions for examination and treatment of common diseases outlined by the Medical Examination and Treatment Administration of the Vietnam Ministry of Health. Incorrect alternatives in the survey were chosen in a way that could allow the doctors to discriminate between possible scenarios, and were revised based on the pilot testing described above. Doctor’s responses were classified as correct or incorrect according to this definition. The first section included a series of 27 questions on common medical emergencies encountered in primary health care, and the responses to this section is the focus of the analyses presented in this article. The questions, and correct answers for this instrument are detailed in Additional File 1. For analytical purposes, these 27 questions are grouped into four categories as follows:

A. Trauma care: 7 questions
B. General critical care:7 questions
C. Cardiovascular emergencies: 6 questions
D. Less common emergencies:7 questions

### Data collection

The questionnaire was paper based and self-administered by the participants, with instructions to independently provide responses to all 27 questions within a period of 15 min, under supervision by training faculty. The same questionnaire was administered across all the eight CME programs. In addition to this clinical component, the analysis used other variables collected for each participant from the training program registration database, including their age, sex, seniority (number of years in medical practice) and province.

### Statistical analysis

Descriptive analysis initially characterized the participating doctors using frequencies by gender, age and seniority by province. These explanatory variables were then categorized as sex (male, female), age (< 35 years, ≥ 35 years), seniority (< 10 years, ≥ 10 years) and location (province), for comparative analysis of test performance.

As described above, each individual question in the survey has been assigned one correct answer as part of the study design. The analysis then involved the following steps. 1) The proportion of doctors who answered with the correct response for each individual question in the survey was calculated. 2) The questions were categorized into 4 broad areas of health knowledge (trauma care, cardiovascular emergencies, general critical care and less common medical emergencies). The number of questions answered correctly in each health knowledge category was then calculated for each doctor. The number of questions answered correctly was then divided by the number of questions in that category to derive a proportion of questions answered correctly in that category for each doctor. The mean proportion of questions answered correctly across the whole sample group in each category was then derived. 3) For each of the health knowledge categories the average proportion of questions answered correctly in that category was then calculated separately by doctor characteristics (age, sex, seniority, and province). Chi-square tests were conducted to assess the statistical significance of differences in the mean proportions of correct answers for each health care question category, and for differences in mean proportions of correct answers by doctor characteristic. A *p*-value of < 0.05 was considered statistically significant for all tests.

## Results

Here we present results regarding the study sample and the proportions of participating doctors, and sub-groups of doctors, who could provide correct answers to questions regarding treatments for common medical emergencies. In total 302 doctors were recruited to the study (Table 1). The majority were male (60.9%), aged more than 35 years (66.0%) and had more than 10 years of medical practice experience (54.3%). The participants were fairly evenly distributed across the three provinces which were assessed for this analysis.

**Table 1:** General characteristics of doctors at CHSs

**Doctor Characteristics**		**n**	**%**
Sex	Male	184	60.9
	Female	118	39.1
Age	< 35 years	133	44.0
	≥ 35 years	169	66.0
Mean age (X ® ± SD) years			38.8 ± 8.2
Seniority	< 10 years	138	45.7
	≥ 10 years	164	54.3
Mean of seniority (X ® ± SD) years			12.3 ± 8.3
Province	Province-1	110	36.4
	Province-2	84	27.8
	Province-3	108	35.8

**Fig. 1:**
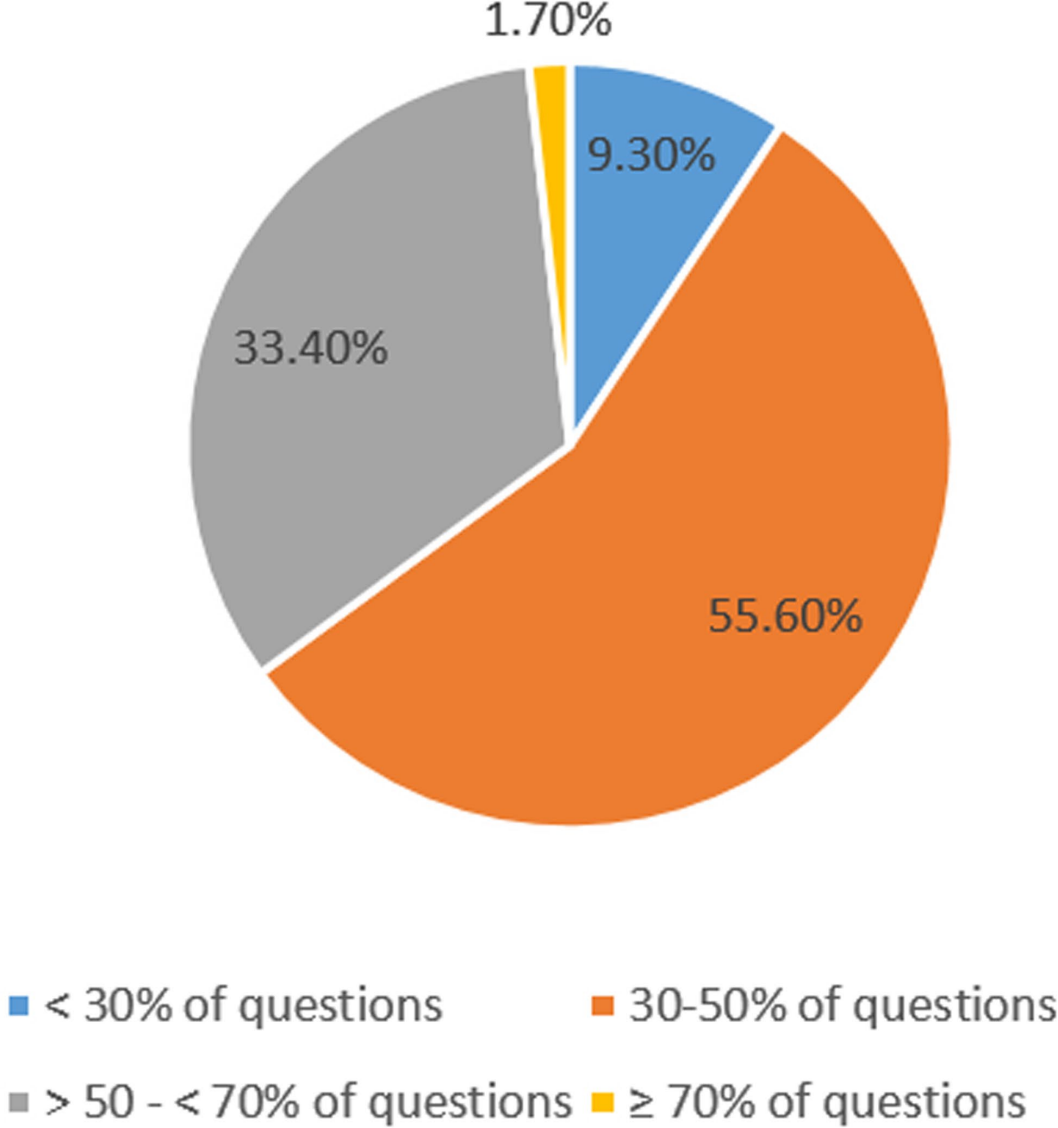
Distribution of overall knowledge by percentage of questions answered correctly

Figure 1 shows the overall distribution of correct answers provided for the health care related questions across the participating doctors. More than half of the sample answered 30–50% of the questions correctly, followed by around a third who answered 50–70% correctly. Less than 2% of doctors provided more than 70% correct responses to the entire question set.

Table 2 presents the proportions of correct answers for each individual question in the survey. As well this table reports the average proportion of questions answered correctly for each broad category of questions. Overall critical care related questions were answered most accurately with an average of 54.6% of questions answered correctly. Individual questions in this category were answered well with only two questions, techniques for Heimlich maneuver (29%) and gastric lavage procedures for hypnotic poisoning (8%) being well below the category average.

**Table 2:** Proportions of doctors providing correct answers regarding treatment for common emergencies

Common emergencies	% of doctors providing correct answers	
	**n**	**%**
**Trauma care (mean proportion of correct answer and SD)**	-	44.70 (19.24)
Principles of general first aid for major fracture	171	56.6
Principles of bone / joint immobilization for fractures	141	46.7
Principle of first aid for open fractures	229	75.8
Criteria/indications for cervical spine immobilization	64	21.2
Choice of equipment to be used for cervical spine immobilization	133	44.0
First aid for patients with pneumothorax under pressure	110	36.4
Method of haemostasis for wounds	97	32.1
**Cardiovascular emergencies (mean proportion of correct answer and SD)**	-	20.53 (13.34)
Criteria of diagnosis necessitating cardiopulmonary resuscitation	78	25.8
Cardiopulmonary resuscitation method (CAB)	72	23.8
The rate of cardiac compression when having only one person for emergency cardiopulmonary resuscitation	56	18.5
First aid for heart attack	174	57.6
First aid on stroke patients, criteria for using drugs to control blood pressure	49	16.2
For first aid patients with stroke, drug of choice for controlling blood pressure	5	1.7
**General critical care (mean proportion of correct answer and SD)**	** -**	54.64 (14.91)
Modalities for safe transportation of patients	277	91.7
Kind of abdominal pain in appendix	270	89.4
Purpose of assessing and triage classification of patients in emergency	96	31.8
First aid for anaphylaxis which occurs during infusion	194	64.2
Signs of airway obstruction	206	68.2
Technic of the Heimlich method	88	29.1
Indications for gastric lavage in patients with hypnotic poisoning	24	7.9
**Less common medical emergencies (mean proportion of correct answer and SD)**	-	44.94 (16.34)
If a femoral fracture occurs, how much blood could be lost	87	28.8
First aid for electric shock	237	78.5
First aid for drowning	248	82.1
First aid for thermal burns	225	74.5
First aid for snake bite	97	32.1
Common signs of heat-stroke	56	18.5

In the trauma care category, questions were answered correctly by an average of 45% of doctors, with the question answered correctly most often addressing first aid for open fractures. Criteria for treating cervical spine issues in accidents was only answered correctly by 21% of doctors. The category with the lowest proportion of correct answers was cardiovascular emergencies with only around 20% of questions correct on average. The question with the least doctors answering correctly in this category, and for the questionnaire as a whole, was regarding the use of medication to control blood pressure in stroke patients which was only correct for 1.7% of doctors. First aid procedures for heart attacks were answered correctly by 57.6% of doctors, the highest proportion in the cardiovascular emergency category. Among less common medical emergencies, the average proportion of correct answers was around 45%. First aid for drowning (82%), electric shock (78.5%) and thermal burns (74.5%) were the questions answered correctly most often.

Table 3 shows the proportion of questions answered correctly by each broad category of questions and by characteristics of the doctors. There were statistically significant differences between question categories, with cardiovascular care questions answered correctly significantly less often than any of the categories. This was true across all doctor characteristic groups. Regarding doctor age, younger doctors answered questions correctly more often across all question categories, with particularly higher proportions for general critical care questions. There were no differences between males and females or doctor seniority groups for any of the question categories. Across provinces, Ha Giang province doctors were significantly less likely to answer questions regarding trauma care or cardiovascular emergencies than the other provinces, but there were no differences for general critical care or less common medical emergencies.

**Table 3:** Proportion of questions answered correctly in each category of emergency care by doctor characteristics

	**Proportion of question correct (%)**			
	**Trauma Care**	**Cardiovascular emergencies**	**General critical care**	**Less common medical emergencies**
Total	44.7	20.5^a^	54.6	44.9
Age				
< 35	45.5	22.0^a^	57.9	47.6
≥ 35	44.2	19.6^a^	52.5	43.2
Sex				
Male	44.6	20.2^a^	53.9	44.5
Female	44.8	21.1^a^	55.8	45.6
Seniority				
< 10 years	44.8	20.9^a^	55.9	45.8
≥ 10 years	44.6	20.2^a^	53.6	44.3
Province				
Province-1	40.8	16.0^a^	55.8	43.2
Province-2	44.7	22.4^a^	52.7	43.4
Province-3	48.7	23.7^a^	54.9	47.9

^a^Indicates statistically significant differences in average proportion of questions answered correctly between question categories, *p*-value is < 0.00001

## Discussion

Overall, our findings reveal low level of knowledge among Vietnamese physicians regarding the most appropriate responses to medical emergencies, and reveal a need for continuing medical education. These results were found in remote mountain provinces, but are likely to apply more broadly in Vietnam. An average of half of the surveyed doctors gave incorrect answers in regard to the most appropriate treatment or response to common medical emergencies. Doctors had the lowest level of knowledge regarding responses to cardiovascular emergencies. This is particularly concerning given stroke is the leading cause of death in the country and other cardiovascular conditions are also in the top ten causes of death [15]. The participants were most likely to answer correctly questions relating to fundamental rules that have not changed or have changed relatively little since they were trained. These included general first aid, pulse check, or identifying signs of airway obstruction. Moreover, their knowledge was potentially based on clinical experience, which likely contributed to their struggle with more theoretical questions such as definitions and updated indications, which were not part of their previous education. Better results were seen in young doctors and recent graduates. Vietnam is a developing country with new international integration processes which are rapidly affecting medical practice with information technology becoming an essential tool for medical staff in daily work as well in updating their practices based on new advances in medicine. Young doctors who are better suited to this approach will have an easier time updating their practices and applying new knowledge into their daily work. Female doctors also scored significantly higher than males.

In our study, questions related to first aid for drowning victims were answered correctly more often than the other questions in the survey (82.1%). The correct knowledge of responses for gastrointestinal poisoning was lower than a previous study which found 91% of doctors knew the correct response [16]. Another study showed that 18.5% of doctors knew management for poison patients [17]. The proportions of doctors in Hanoi – the capital of Vietnam—who answered correctly about airway obstruction and *cardiopulmonary resuscitation*were also higher than in our study (67.9% vs. 29.1%; 31.4% vs. 23.8%) [16]. In this Hanoi study, doctors who were under 35 years of age and who had worked for less than 10 years were more knowledgeable about the management of airway obstruction and anaphylaxis than the rest of the doctors. This proportion is higher than that found in a recent study on injury accidents in Binh Dinh province (66.9%) [18].

A low proportion of participating doctors answered questions correctly regarding circulatory arrest emergency responses (23.8%). A study on out-of-hospital cardiac arrest in Hanoi in 2018, 55.8% of patients were rescued, but only 7.6% re-established circulation at the scene and the survival rate to the hospital emergency department was 4.5% [19]. Another study in Botswana also showed same results, knowledge of correct cardiopulmonary resuscitation was held by 48% of doctors, after training the rate increased by 26.4% [20]. The correct knowledge of doctors about first aid for patients bitten by snakes was only found in 32.1% of doctors, a study in Nigeria showed a higher rate, most of them had full knowledge of the clinical features of snakebites (62.3%), first aid treatment (75.7%), but only at an average level [21]. Another study in India on snakebite management showed that over 90% of health facilities are primary care centers run by general practitioners [22]. Despite having basic equipment for successfully treating snakebite including antivenom, most of the doctors do not treat snakebite, simply because doctors lack confidence in diagnosing and treating for patients [22].

The majority of doctors gave correct responses to the question regarding how to manage patients with anaphylaxis (64.2%), a similar study in Scotland found this rate to be higher, 90% of the doctors interviewed knew to use adrenaline as first-line treatment, 36% of physicians had experience in managing at least one out-of-hospital anaphylaxis, male physicians had better knowledge than female physicians (*p* < 0.05), doctors with younger age have higher knowledge about the management of patients with anaphylaxis (*p* < 0.05) [23]. The percentage of doctors with correct knowledge about first aid for burn patients is relatively high (accounting for 74.5%). In a study in Turkey showing lower results, only 22% of doctors knew how to properly classify burns, only 4% of doctors chose the right first aid method for burns and 32% using modern first aid methods [24]. Another study in Western Australia reported that 50% of patients received incorrect first aid for burns, the rate of correct answers was only 19%, male doctors had better knowledge than female doctors (*p *< 0.05), younger doctors have lower knowledge of burn first aid than the other group (*p*> 0.05) [25].

### Strengths and limitation of the study

The strengths of this study lie in its relatively large sample of doctors working in CHSs in remote mountainous areas of Vietnam. The questionnaire on knowledge of common emergencies in the community was also designed in a relatively detailed manner. The study also has some limitations. Findings are restricted to the provinces studied, which may have unique characteristics as compared to rest of Vietnam, hence not generalizable. Additionally, the doctors provide insight into their knowledge of common emergencies in their own contexts. It is not possible to extrapolate to CHSs doctors in rural or urban areas, where conditions are different. No other information was available regarding incidence of common emergencies faced by these doctors, which could have helped to interpret the relatively low scores. That is, because the doctors do not face these emergencies very often in their regular practice, they may not be remembering all the details about the correct responses. Also, the participants were given a relatively short time to complete the questionnaire (15 min for 27 questions) so doctors may not have quick recall, so the pre-test results may not be a true picture of baseline prior knowledge of physicians. Study subjects interviewed with self-filled questionnaires before taking the course may have produced inaccurate results participants could consult each other. This was prevented by having supervision of the instructors during the completion of the forms, avoiding mutual exchanges. This study investigates the knowledge of common emergencies. Further studies are needed to evaluate practice skills of doctors working at CHSs.

## Conclusion

The findings reported here show that the doctors who participated in the study have relatively low knowledge on common emergencies, particularly for cardiovascular care questions. The results also support the need for continuing medical education to improve doctors’ knowledge, who are mostly practicing in resource limited remote settings. Based on our results, we recommend several next steps. First, it is necessary to conduct more targeted continuing medical education, the organizer should pay more attention to male doctors, and the older medical staff, change the educational method, concentrate more on training about common emergencies to make them comfortable with receiving updated knowledge. Doctors over 35 years old and doctors with more than 10 years of seniority need to be prioritized in these training courses, compared to other groups.

## Supplementary Information

**Additional file 1.**

## Data Availability

Data are available on request from the study authors.
