# Supplementary material for: Continuing medical education programs for primary care physicians from remote locations of Vietnam: a needs assessment
Source: BMC Med Educ. 2022 Apr 13;22:279. doi: 10.1186/s12909-022-03336-4 (PMC9008997; doi:10.1186/s12909-022-03336-4)
Supplement: Supplementary file 1 — Additional file 1. [file 12909_2022_3336_MOESM1_ESM.docx]

**Supplementary Table1: Question-items, original questions, response options and correct answer(s) in the questionnaire**

| **Question-items** | **Original questions** | **Response options** | **Correct answer(s)** |
| --- | --- | --- | --- |
| **Trauma care** | | | |
| Principles of general first aid for major fracture |  |  |  |
| Principles of bone / joint immobilization for fractures |  |  |  |
| Principle of first aid for open fractures |  |  |  |
| Criteria/indications for cervical spine immobilization |  |  |  |
| Choice of equipment to be used for cervical spine immobilization |  |  |  |
| First aid for patients with pneumothorax under pressure |  |  |  |
| Method of haemostasis for wounds |  |  |  |
| **Cardiovascular emergencies (mean proportion of correct answer and SD)** | | | |
| Criteria of diagnosis necessitating cardiopulmonary resuscitation |  |  |  |
| Cardiopulmonary resuscitation method (CAB) |  |  |  |
| The rate of cardiac compression when having only one person for emergency cardiopulmonary resuscitation |  |  |  |
| First aid for heart attack |  |  |  |
| First aid on stroke patients, criteria for using drugs to control blood pressure |  |  |  |
| For first aid patients with stroke, drug of choice for controlling blood pressure |  |  |  |
| **General critical care (mean proportion of correct answer and SD)** | | | |
| Modalities for safe transportation of patients |  |  |  |
| Kind of abdominal pain in appendix |  |  |  |
| Purpose of assessing and triage classification of patients in emergency |  |  |  |
| First aid for anaphylaxis which occurs during infusion |  |  |  |
| Signs of airway obstruction |  |  |  |
| Technic of the Heimlich method |  |  |  |
| Indications for gastric lavage in patients with hypnotic poisoning |  |  |  |
| **Less common medical emergencies** | | | |
| If a femoral fracture occurs, how much blood could be lost |  |  |  |
| First aid for electric shock |  |  |  |
| First aid for drowning |  |  |  |
| First aid for thermal burns |  |  |  |
| First aid for snake bite |  |  |  |
| Common signs of heat-stroke |  |  |  |
